# Habitat Structure Outweighs Monastic Legacy in Shaping Bird Assemblages

**DOI:** 10.3390/ani16101534

**Published:** 2026-05-17

**Authors:** Łukasz Jankowiak, Kinga Piórkowska, Michał Polakowski, Sebastian Michałowski, Michał Szkudlarek

**Affiliations:** 1 Department of Ecology and Anthropology, Institute of Biology, University of Szczecin, Wąska 13, 71-899 Szczecin, Poland; 2 West Pomeranian Nature Society, Wąska 13, 71-899 Szczecin, Poland

**Keywords:** sacred natural sites, cultural landscapes, land-use legacy, bird assemblages, landscape heterogeneity, farmland biodiversity

## Abstract

Sacred natural sites are often considered potential refugia for biodiversity in human-dominated landscapes, but their effects can be confounded by present-day habitat structure. We tested whether extant Cistercian monasteries in western Poland influence breeding-bird assemblages at two spatial scales by conducting standardized 5 min point counts during two visits at 234 stations across 23 plots and comparing Cistercian plots with environmentally matched Control and Post-Cistercian plots. We recorded 133 breeding-bird species, numerically dominated by widespread farmland and synanthropic taxa. Neither plot category nor station placement within versus outside monastery grounds explained variation in Shannon diversity or rarefied species richness. In contrast, both diversity metrics increased with contemporary landscape complexity, especially along gradients from arable land toward grasslands and urban habitats and with increasing heterogeneous agriculture, while community composition was significantly associated with current landcover structure. These findings indicate that present-day habitat structure, rather than monastic legacy, is the main driver of breeding-bird diversity in this system. Conservation and land-use policy in agricultural regions should therefore prioritize the maintenance and restoration of heterogeneous landscapes, including mosaics of semi-natural habitat elements.

## Simple Summary

Historic religious sites are often thought to help protect wildlife in landscapes heavily changed by people, but it is not always clear whether this reflects their historical impact on biodiversity or simply the fact that the surrounding habitat remains suitable today. We studied breeding birds around Cistercian monasteries in western Poland to test whether these sites still have a unique influence on bird diversity. We counted birds at 234 survey points in 23 study areas, including active monastery sites, similar sites without monasteries, and places where monasteries once existed. In total, we recorded 133 bird species, mostly common farmland and town-associated birds. We found that bird diversity was not higher simply because a site was linked to a monastery, nor because survey points were located inside monastery grounds. Instead, bird diversity was greater in areas with more varied modern landscapes, especially where open fields were mixed with grasslands, built-up areas, and small-scale farming habitats. This shows that current habitat structure matters more than historical monastic legacy for breeding birds in this system. Therefore, protecting and restoring diverse agricultural landscapes is likely to be more effective for bird conservation than relying on historical land use alone.

## 1. Introduction

Sacred natural sites (SNSs) may receive particular forms of protection or stewardship associated with religious, spiritual, or cultural beliefs and traditions, and are sometimes recognized as components of conservation networks and biocultural heritage (e.g., [1,2]). However, bird assemblages in such sites are unlikely to be shaped by cultural or religious factors alone. Instead, local avifauna is typically influenced by a combination of current habitat structure, vegetation complexity, availability of nesting and foraging sites, management intensity, disturbance, patch size, and the composition of the surrounding landscape. While spiritual, cultural, and religious factors are not the sole determinants of the ecological quality of such areas, they may contribute to it by fostering various forms of stewardship and care. Across regions and taxonomic groups, SNSs may function as refugia that maintain habitats, species, and ecological processes within human-dominated landscapes, often through long-term local stewardship and social norms that limit resource exploitation [3,4,5].

In most empirical studies, SNSs are operationalised as relatively small, delimited patches (e.g., sacred groves, churchyards, monastery grounds) embedded in broader production landscapes [5]. In European cultural landscapes, structurally diverse green enclaves such as churchyards and cemeteries can support bird assemblages comparable to other urban green spaces, highlighting the role of patch-level vegetation structure and management in shaping local diversity [6,7]. Accordingly, variation in biodiversity among sacred patches is often mediated by local vegetation structure and management as well as by the surrounding landscape context. Their biodiversity value may vary from site-level protection and vegetation structure within the sacred patch itself to wider landscape legacies linked to long-term stewardship and land-use practices surrounding the site. This is particularly relevant in Central and Eastern Europe, including Poland, where agricultural land covers approximately 60% of the country’s area [8], making rural landscapes a dominant component of the environment. In such systems, biodiversity patterns—especially in bird assemblages—are strongly linked to farmland habitats and their structural heterogeneity, although these have declined under agricultural intensification [9,10].

Long-lived institutions (e.g., monasteries) have historically shaped both land use (e.g., settlement, water management) and animal distribution patterns (e.g., via translocations), creating potential for persistent ecological legacies. Among Christian monastic orders, Cistercians are especially strongly associated with large-scale landscape transformation, notably through granges, mills, drainage, canals, and fishpond systems that modified local hydrology and land cover, often leaving durable traces in the landscape [11]. In addition to these historical transformations, monastic influence may also persist through the present-day stewardship of monastery grounds, including the maintenance of gardens, parks, tree cover, ponds, orchards, and other semi-natural elements that can provide fine-scale refugia within otherwise intensively managed landscapes [12]. Such legacies may be detectable today as differences in community composition or diversity relative to comparable sites without monastic influence, though isolating this effect may be challenging given the confounding influences of the current habitat structure [13,14].

Here, to test SNS effects on biodiversity, we use Cistercian monasteries and bird assemblages, because Cistercians were among the monastic orders most strongly associated with large-scale landscape transformation, and because bird assemblages respond sensitively to habitat structure [15] and can therefore capture such legacy effects. We focus on the spatial and historical footprint of monastic land use as a measurable proxy of potential long-term influence. This approach does not directly capture contemporary religious beliefs, practices, or governance systems, but rather tests whether any residual effect of historical land-use legacy can be detected after accounting for the current landscape structure. For this, first, we test a plot-scale legacy by comparing whole 2 km radius plots centred on extant Cistercian monasteries with environmentally matched (i) Control plots, and (ii) historical (non-extant) Cistercian plots. Second, we test a site-scale SNS effect by contrasting point-count stations located within monastery grounds against comparable stations beyond the monastery grounds. Because control plots were selected to match the landscape composition of the monastery plots (resulting in many within river valleys and other high-value landscapes), any Cistercian–Control plot differences would indicate effects beyond general habitat quality. We therefore hypothesized that (i) bird diversity differs among Cistercian, Post-Cistercian, and Control plots (plot-scale SNS effect), (ii) stations within monastery grounds show higher avian diversity than other stations (site-scale SNS effect), and (iii) independent of the tested SNS effect, diversity increases with current habitat heterogeneity and along gradients from arable land toward grasslands and urban habitats.

## 2. Materials and Methods

### 2.1. Study Area, Plot Definition, and Site Selection

**Figure 1:**
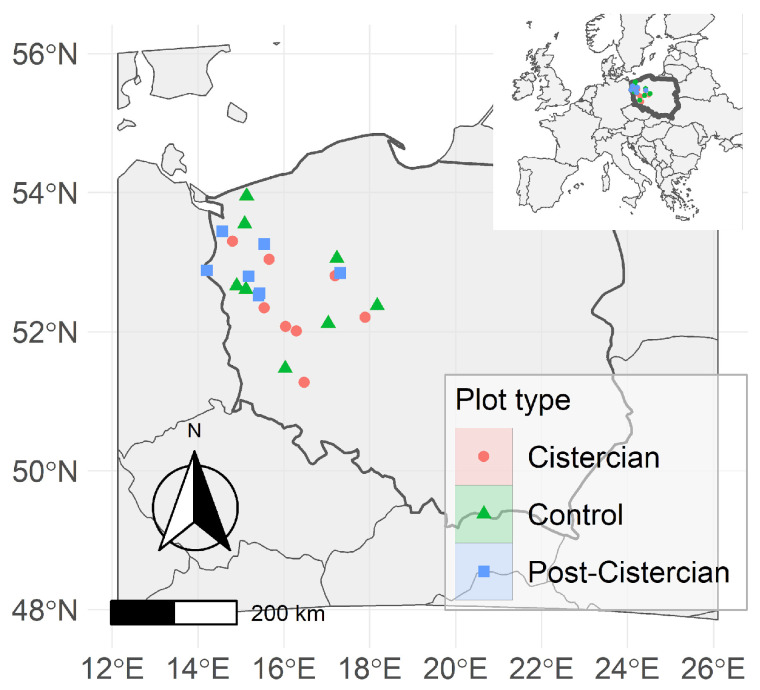
Location of the surveyed Cistercian (*n* = 8), Control (*n* = 8), and Post-Cistercian (*n* = 7) plots within Poland.

Bird surveys were conducted in western Poland within four voivodeships: Zachodniopomorskie (West Pomeranian), Lubuskie (Lubusz), Wielkopolskie (Greater Poland), and Dolnośląskie (Lower Silesian). The eight focal extant Cistercian monastery plots (factor level Plot = Cistercian), shown in Figure 1, were centred on the following monasteries: Kołbacz (53.301626 N, 14.813274 E), Bierzwnik (53.039442 N, 15.659944 E), Paradyż/Jordanowo (52.336906 N, 15.545220 E), Lubiąż (51.262411 N, 16.468304 E), Obra (52.071356 N, 16.056232 E), Przemęt (52.009964 N, 16.292169 E), Ląd (52.203920 N, 17.893131 E), and Wągrowiec (52.804852 N, 17.191842 E). Each focal plot was defined with a 2 km radius buffer around the monastery, within which landscape composition was quantified from CORINE Land Cover 2018 by calculating habitat-category percentages. CORINE Land Cover 2018 was used as a standardized dataset ensuring consistent classification of land-cover types across the study area. The dataset has a minimum mapping unit of 25 ha and a spatial resolution of 100 m [16], which is appropriate for capturing landscape-scale habitat patterns. The 2 km radius spatial extent was chosen to reflect the expected scale of Cistercian legacy effects, which likely operated beyond the monastery grounds through estate-based land-use and hydrological modifications. Accordingly, we treated the entire 2 km plot, including stations located near its edges, as potentially lying within the spatial footprint of historical Cistercian influence.

To select control plots (Plot = Control) with comparable landcover composition but free from potential monastery influence, we generated a large set of candidate random plot centres within the study region after applying an exclusion mask based on 20 km radius buffers around all extant and historical Cistercian localities. This ensured that candidate controls were spatially separated from any Cistercian influence, including areas where monasteries no longer exist. Each candidate control centre was then buffered by 2 km, and the same CORINE-derived habitat percentages were computed. Control plots were retained by selecting those whose landcover composition most closely matched the landcover composition of the eight monastery plots (i.e., using the multivariate landscape composition of the monastery buffers as the target reference for matching), thereby producing eight controls that were environmentally comparable to the monastery plots while remaining outside the 20 km exclusion zones. Similarly, seven plots centred on historically documented but currently non-existent monastery localities were treated as a separate plot category (Plot = Post-Cistercian) and were likewise defined with 2 km buffers. The localisation of the surveyed plots is shown in Figure 1.

Within each selected plot (Cistercian, Control, and Post-Cistercian), we generated a set of candidate point-count stations by uniform random placement inside the 2 km buffer while enforcing a minimum spacing of 200 m among the stations. This produced a surplus of potential stations (40 per plot), from which 10–11 stations per plot were selected based on accessibility and feasibility, while maintaining the intended spatial dispersion. Each surveyed station was uniquely labelled by site_name (e.g., Bierzwnik1, Bierzwnik10), nested within the plot identity main_area (e.g., Bierzwnik), and plot category Plot (e.g., Cistercian, Control, Post-Cistercian).

### 2.2. Bird Counts

Birds were surveyed using standardized 5 min point counts with two visits per station during the breeding season. The surveys were carried out in 2024 and 2025, with different plots surveyed in each year for logistical reasons, as the total number of plots and point-count stations could not be completed within a single breeding season. Seasonal variability was not analyzed; instead, its potential influence was minimized by applying the same field protocol and the same predefined survey windows in both years. The first visit was conducted between 10 April and 15 May and the second between 16 May and 30 June, with visits separated by at least four weeks. Counts were performed in the morning (before 10:00 in April and before 9:00 in May–June) under suitable weather conditions. All birds detected visually or by sound were assigned to species and distance at first detection (0–25 m, 25–100 m, >100 m, and birds in flight), applying conservative rules to avoid double-counting moving individuals; individuals not confidently identified to species were not included.

### 2.3. Habitat Characterization of Surveyed Count Stations and Statistical Analyses

All statistical analyses were restricted to point-count stations that were surveyed exactly twice. For these stations, geographic coordinates (latitude/longitude) and survey metadata were consolidated at the station level (site_name), retaining plot category (Plot: Cistercian, Control, Post-Cistercian), plot identity (main_area), observer identity (count_observers), and the timing of each of the two visits.

**Figure 2:**
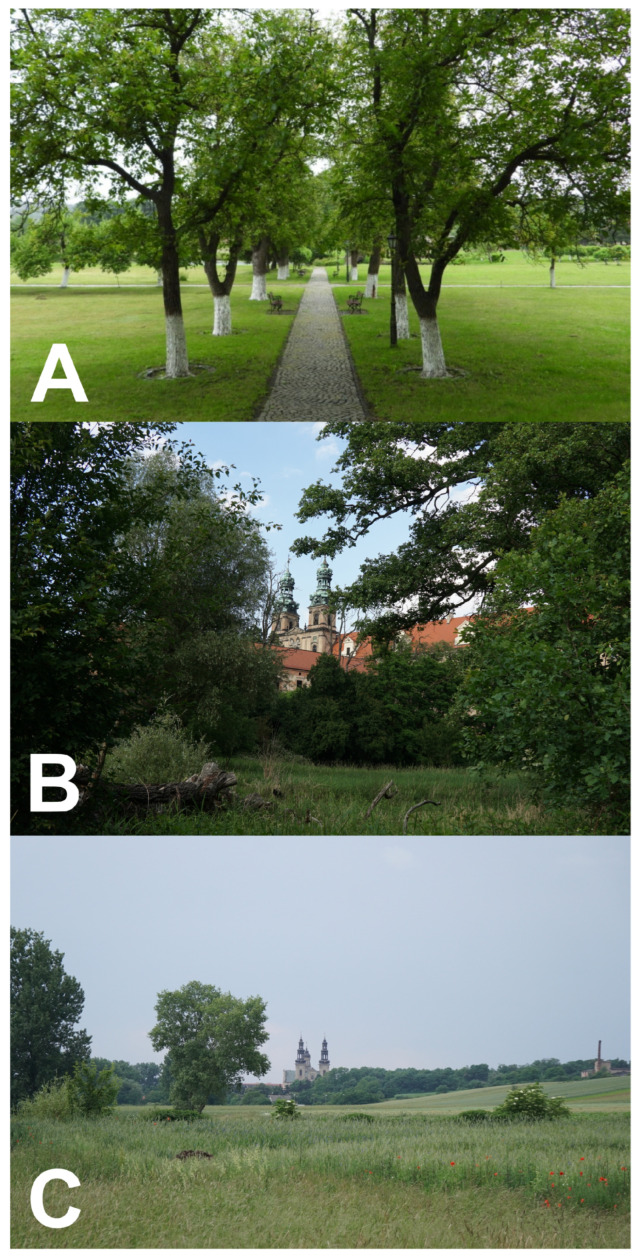
Representative examples of Cistercian monastery study sites in western Poland. (**A**) Orchard within the monastery grounds in Paradyż; (**B**) semi-natural vegetation and monastery surroundings in Lubiąż; (**C**) agricultural landscape surrounding the monastery in Ląd. Photographs: (**A**) ŁJ; (**B**,**C**) MS.

Following the final field selection of 10–11 stations per plot, habitat composition was quantified specifically for the surveyed stations. For each station, land cover within a 2 km radius was summarized using CORINE Land Cover 2018, producing proportional cover of major habitat classes (including Arable land, Forest, Meadows_grassland, Heterogeneous_agri, Urban, Inland_wetlands, and Inland_water). This spatial scale was chosen to capture the broader landscape context influencing bird assemblages, rather than the home ranges of individual birds. Habitat summaries were joined to the station-level bird-count dataset by site identifier. Where a habitat class was absent from a buffer, its value was set to zero. Forest cover was defined as the sum of CORINE forest plus urban green space (Urban_green), thereby capturing tree-dominated habitats across both natural and human-modified landscapes. The averaged habitat composition for each three plot type categories is shown in Supplementary Materials Table S1. Monastery grounds typically included park-like greenery characterized by mature trees, orchards (Figure 2A), and semi-natural vegetation structures (Figure 2B), embedded within a broader agricultural landscape context (Figure 2C), which together influence local habitat conditions.

Each station was additionally classified with a binary variable monastery_direct, coded 1 for stations located within the monastery garden/grounds (i.e., within the area enclosed by the monastery walls or fence) and 0 otherwise (including other stations within monastery plot, all Control plots, and post-Cistercian plots).

Bird community data were compiled by aggregating species abundances within each station across the two visits. Species-by-station community matrices were constructed with stations as rows and species as columns (abundances). Diversity metrics were calculated using the vegan package (version 1.13-2) [17] in the R environment (version 4.1.3) [18]. For each station, we derived total abundance, observed species richness, and Shannon diversity; Shannon diversity was used as the principal response variable in the mixed-effects models.

To address collinearity among geographic and habitat predictors, we first inspected variance inflation factors and pairwise correlations among candidate covariates (longitude, latitude, and the main CORINE habitat proportions). We then applied principal component analysis to a centred and scaled predictor matrix comprising longitude, latitude, Arable, Forest, Meadows_grassland, Heterogeneous_agri, and Urban. The first four principal components were retained and interpreted as composite gradients in landscape structure; these axes were carried forward as predictors and labelled arable_to_urban_PC1, arable_to_grass_PC2, urban_to_grass_forest_PC3, and Heter_agri. This approach was used to avoid multicollinearity among correlated land-cover predictors and to summarize the main environmental gradients in a parsimonious and interpretable form. Correlations between the retained PC axes and the original covariates are shown in Supplementary Materials Figure S1.

We fitted two linear mixed-effects models (REML): one with Shannon diversity (H’) as the response and a second with rarefied species richness as the response. In both models, fixed effects included plot category (Plot: Cistercian, Control, Post-Cistercian), the binary monastery_direct predictor (inside vs. outside of the monastery grounds), and the four PCA-derived landscape gradients (arable_to_urban_PC1, arable_to_grass_PC2, urban_to_grass_forest_PC3, Heter_agri). To account for the nested sampling design and repeated field implementation across areas and observers, we included a random intercept for main_area. In addition, the selected models explicitly accounted for observer effects by including observer-specific random effects: an observer intercept and observer-specific slopes for Plot, allowing the estimated effect of plot category to vary among observers (with slope terms treated as uncorrelated).

### 2.4. Ordination of Community Composition

To examine how bird community composition varied with current land cover, we used constrained ordination in vegan. Species-by-station abundances (summed across the two visits per station) were Hellinger-transformed (decostand, method = “hell”). We attempted Detrended Correspondence Analysis (DCA) to assess gradient lengths; because the first-axis gradient length was non-finite, we proceeded with Redundancy Analysis (RDA) rather than Canonical Correspondence Analysis (CCA). The full RDA model included the main CORINE habitat proportions (Arable, Forest, Meadows_grassland, Heterogeneous_agri, Urban). Model significance was evaluated with 999 permutations under a reduced model, testing the overall constrained model as well as significance by axis and by term (anova.cca with by = “axis” and by = “margin”).

## 3. Results

### 3.1. Bird Assemblage Composition

**Figure 3:**
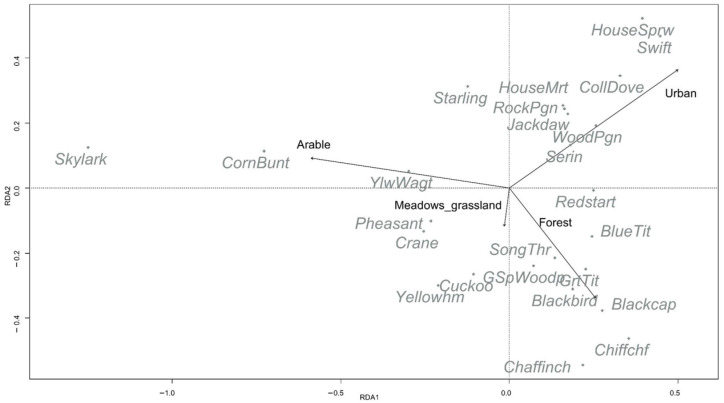
Redundancy analysis (RDA) ordination of breeding bird assemblages constrained by landscape composition. Only 25 of the most numerous species are shown.

The bird assemblage comprised 133 species and was numerically dominated by a small set of common farmland and synanthropic taxa. The most abundant species was European Starling *Sturnus vulgaris* (17.16%, *n* = 1312), followed by Eurasian Skylark *Alauda arvensis* (6.93%, *n* = 530), House Sparrow *Passer domesticus* (6.66%, *n* = 509), Common Wood Pigeon *Columba palumbus* (5.38%, *n* = 411), and Barn Swallow *Hirundo rustica* (3.39%, *n* = 259). Together, these five species accounted for 39.52% of all individuals. The next most abundant species were Corn Bunting *Emberiza calandra* (3.01%, *n* = 230), Common Blackbird *Turdus merula* (2.84%, *n* = 217), Eurasian Blackcap *Sylvia atricapilla* (2.56%, *n* = 196), Eurasian Collared Dove *Streptopelia decaocto* (2.55%, *n* = 195), and Rock Dove/Feral Pigeon *Columba livia* (2.42%, *n* = 185); all these ten most abundant species comprised 52.90% of all records. Patterns in the ordination (Figure 3) were consistent with this dominance structure, separating open-country farmland species (e.g., skylark, corn bunting) from urban-associated taxa (e.g., house sparrow, swift, collared dove, feral pigeon). The counts of all recorded species are provided in Supplementary Materials Table S2.

Bird records from monastery gardens/grounds comprised 242 individuals (3.16% of all detections) and were dominated by synanthropic and ecotone-associated species (Supplementary Materials Table S3). The most abundant taxa were Rock Dove/Feral Pigeon (12.40%, *n* = 30), Common Wood Pigeon (10.74%, *n* = 26), European Starling (9.92%, *n* = 24), Eurasian Collared Dove (4.13%, *n* = 10), and Eurasian Skylark (3.72%, *n* = 9); together, these five species accounted for 40.91% of records. Eurasian Blackcap and Common Chaffinch *Fringilla coelebs* each contributed 3.31% (*n* = 8), and Great Tit *Parus major* contributed 2.89% (*n* = 7). Several urban-associated species (each 2.48%, *n* = 6) were also prominent, including House Sparrow *Passer domesticus*, Common Swift *Apus apus*, European Goldfinch *Carduelis carduelis*, Common Chiffchaff *Phylloscopus collybita*, European Greenfinch *Chloris chloris*, and European Serin *Serinus serinus*. Relative to other stations, those located within monastery areas showed markedly higher proportional contributions of pigeons/doves (e.g., *C. livia* 12.40% vs. 2.09%; *C. palumbus* 10.74% vs. 5.20%), whereas open-farmland species were underrepresented (e.g., starling 9.92% vs. 17.40%; skylark 3.72% vs. 7.04%; house sparrow 2.48% vs. 6.80%).

### 3.2. Ordination (RDA) of Bird Community Composition

Community composition showed a significant association with current land cover in the constrained ordination (RDA; overall model: F = 7.13, df = 5228, *p* = 0.001). The environmental predictors explained 13.5% of total community variation (R^2^ = 0.131; adj. R^2^ = 0.116). The first two constrained axes captured most of the constrained structure (RDA1 eigenvalue = 0.058, RDA2 = 0.0245), together representing 90.3% of the constrained variance (RDA1 alone 63.6%). Axis permutation tests supported both RDA1 and RDA2 (all *p* = 0.001).

The ordination separated stations along an arable-to-urban gradient on RDA1 (Arable negative vs. Urban positive), with Forest also loading toward the positive side, and Meadows/grassland loading mainly on negative RDA2. Species scores reflected these gradients: open-land farmland species such as Eurasian Skylark *Alauda arvensis* and Corn Bunting *Emberiza calandra* were associated with higher arable cover (negative RDA1), whereas urban-associated species (House Sparrow *Passer domesticus*, Common Swift *Apus apus*, Eurasian Collared Dove *Streptopelia decaocto*, feral pigeon *Columba livia*) aligned with the Urban vector (positive RDA1 and RDA2). Woodland-associated taxa (e.g., Common Chiffchaff *Phylloscopus collybita*, Chaffinch *Fringilla coelebs*, Eurasian Blackcap *Sylvia atricapilla*, Great Tit *Parus major*, Blue Tit *Cyanistes caeruleus*) were positioned toward the Forest direction (positive RDA1, negative RDA2). In marginal permutation tests (controlling for the other predictors), Arable (*p* = 0.001), Urban (*p* = 0.001), and Meadows_grassland (*p* = 0.001) were supported, whereas Forest showed weak evidence (*p* = 0.068). Backward stepwise selection indicated that Heterogeneous_agri was not supported (*p* = 0.281).

### 3.3. Biodiversity Measurement Analysis

Across the 234 point-count stations (23 plots; four observers), bird diversity metrics were primarily explained by current landscape structure rather than by plot category or monastery-related predictors.

**Figure 4:**
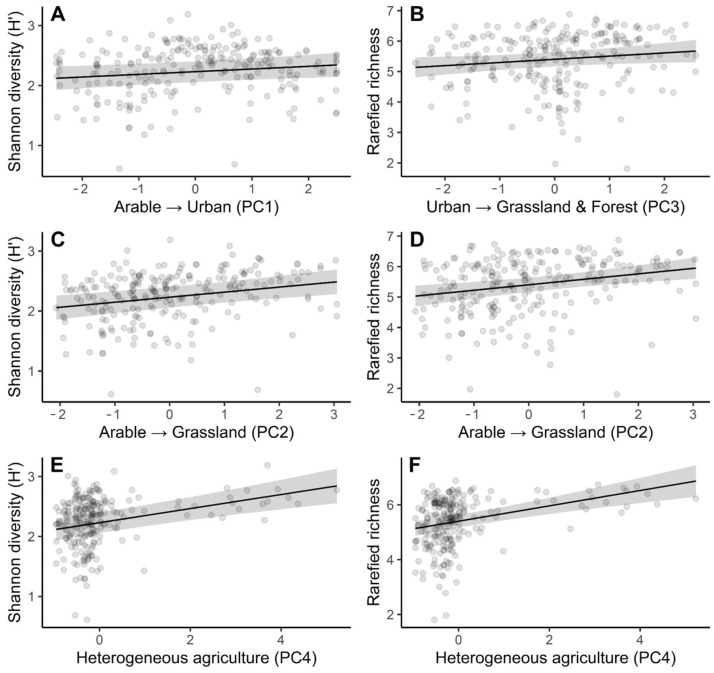
Relationships between breeding bird diversity and PCA-derived landscape gradients. Shannon diversity (H′) is shown for (**A**) Arable → Urban (PC1), (**C**) Arable → Grassland (PC2), and (**E**) Heterogeneous agriculture (PC4). Rarefied species richness is shown for (**B**) Urban → Grassland and Forest (PC3), (**D**) Arable → Grassland (PC2), and (**F**) Heterogeneous agriculture (PC4). Points represent point-count, and solid lines indicate fitted relationships, whereas shaded bands show 95% confidence intervals.

In the mixed-effects model for Shannon diversity, plot category did not differ detectably among Cistercian, Control, and Post-Cistercian plots (overall effect of Plot: F_2,4.92_ = 0.52, *p* = 0.624). Relative to Cistercian plots (reference level), Shannon diversity showed no evidence of difference in Control plots (β = 0.096 ± 0.124 SE, t = 0.77, *p* = 0.491) or Post-Cistercian plots (β = −0.012 ± 0.074, t = −0.17, *p* = 0.869). The binary monastery-related predictor (monastery_direct) was also unrelated to Shannon diversity (β = −0.046 ± 0.124, t = −0.37, *p* = 0.710; overall effect of monastery: F_1,223.5_ = 0.52, *p* = 0.624). Full model parameter estimates are provided in the Supplementary Materials (Table S4). By contrast, Shannon diversity increased along the PCA-derived gradients interpreted as transitions from arable land toward more urban cover (arable_to_urban_PC1: F_1,150.1_ = 5.20, β = 0.0445 ± 0.0195, t = 2.28, *p* = 0.024) and from arable land toward grassland (arable_to_grass_PC2: F_1,210.9_ = 14.80, β = 0.0840 ± 0.0218, t = 3.85, *p* < 0.001). Shannon diversity also showed a strong positive association with the heterogeneous agriculture axis (Heter_agri: F_1,223.1_ = 25.41, β = 0.117 ± 0.0233, t = 5.04, *p* < 0.001), whereas the urban-to-grass/forest gradient (urban_to_grass_forest_PC3) was not supported as a predictor (β = 0.0275 ± 0.0226, t = 1.22, *p* = 0.225). These positive relationships are illustrated in Figure 4A (PC1), Figure 4C (PC2), and Figure 4E (PC4).

Rarefied species richness showed the same lack of evidence for plot-category differences (overall Plot effect: F_2,3.84_ = 0.063, *p* = 0.940) and for monastery_direct (F_1,221.2_ = 0.003, *p* = 0.959), indicating no sacred natural site effect in this aspect. Full model parameter estimates are provided in the Supplementary Materials (Table S5). Rarefied richness increased with urban_to_grassland/forest gradient (PC3: F_1,174.7_ = 4.21, *p* = 0.0417), arable-to-grassland gradient (PC2: F_1,125.7_ = 12.05, *p* < 0.001), and heterogeneous agriculture (PC4: F_1,223.5_ = 27.58, *p* < 0.001). These positive trends are depicted in Figure 4B,D,F, respectively. The arable-to-urban gradient (PC1) was not supported for rarefied richness (F_1,83.4_ = 0.93, *p* = 0.338).

## 4. Discussion

As opposed to our predictions, neither plot category nor direct association with Cistercian monasteries was related to differences in bird diversity or species richness. This negative result is meaningful as it may reflect socio-ecological decoupling, i.e., a situation in which religious values are not translated into conservation outcomes due to factors such economic pressures, institutional changes, or cultural shifts. Therefore, the apparent biodiversity value of sacred natural sites should not be taken for granted but instead evaluated in relation to present-day habitat structure and management practices. Likewise, diversity consistently increased following a gradient from arable land toward grasslands and urban areas. The bird assemblages were dominated by a small set of widespread farmland and synanthropic species, while overall community composition and diversity were primarily shaped by current landscape structure.

Historically, Christian monastic communities have been associated with landscape transformation and land management practices [11]. Care for the surrounding environment was often expressed through stewardship practices, which in practice manifested in the maintenance of gardens, green spaces, and park-like landscapes in ecologically favourable conditions. Such stewardship by clergy, regardless of the monastic order, can maintain small but valuable habitat patches protected by cultural and social norms. These patches occur worldwide across diverse ecosystems, often in regions of high biodiversity, and are recognized as having the potential to complement formal conservation strategies [3,19,20]. Our findings contribute to the growing body of literature showing that the biodiversity value of sacred natural sites is highly context-dependent. While many studies report positive effects of SNS on biodiversity, these are often mediated by local habitat structure, management practices, and broader landscape context rather than by their cultural or historical status per se [5,19]. More broadly, cultural and social drivers such as religion shape ecological patterns indirectly through their effects on landscape structure and human management decisions [21]. In this sense, our results support the view that SNS effects cannot be assumed a priori but must be evaluated relative to contemporary ecological conditions.

In our study, the monastery grounds indeed exhibited relatively high biodiversity; however, they did not differ in species richness or community composition from control sites of similar environmental character. This suggests that, while monastery-associated stewardship may contribute to maintenance of habitat patches, it does not necessarily result in higher bird diversity than that found in comparably heterogeneous landscapes. Untouched and habitat-diverse areas can therefore be as valuable, or even more so, than those actively managed by humans, irrespective of the presence of monastic land-use legacies.

Our findings further indicate that the observed patterns of bird assemblages primarily reflect the intrinsic properties of habitats themselves, rather than direct human impacts. Although a guild-based analysis of forest specialists could be informative, our study focused on whole-community responses because the main habitat gradients were associated with arable land, grasslands, heterogeneous agriculture, and built-up areas rather than continuous forest. Habitat heterogeneity provides a broad range of ecological niches, supporting greater species richness and offering diverse opportunities for foraging, resting, and breeding [9,10,15]. In western Poland, homogeneous farmland typically constitutes a relatively poor ecosystem type, although field margins/hedgerows serve as enclaves of avian biodiversity [22]. As a result, despite its generally low species richness, the agricultural landscape retains specific ecological values.

Overall, our results suggest that more complex habitat structure (typical of ecologically richer landscapes such as grasslands and heterogeneous urban areas) is the main factor shaping the diversity of bird assemblages. Urban and grassland areas have an advantage over farmland due to their greater mosaicity and the diversity of microhabitats available to birds. For example, in urban environments, it has been shown that a mix of built structures, gardens, parks, hedgerows, and tree lines support substantially higher bird diversity [23]. Similarly, grasslands offer a complex habitat structure, including open areas, wetlands, and extensive shrub and tree cover, which sustain a more diverse assemblage of birds [24]. Recent global analyses suggest that cemeteries can be at least as valuable as other urban green spaces for biodiversity, reinforcing the conservation relevance of culturally protected green areas within human-dominated landscapes [25]. Human management, even when long-lasting and conservation-oriented, appears to play a secondary role relative to the intrinsic ecological value stemming from the habitat structure.

From a conservation perspective, our results highlight that maintaining and restoring habitat heterogeneity within agricultural landscapes is more effective for supporting bird diversity than relying on historical land-use legacies alone (as a sole explanatory factor). This includes the preservation of small-scale habitat elements such as field margins, hedgerows, grasslands, and mixed land-use mosaics, which provide complementary resources for breeding birds. Importantly, in the context of SNS, conservation efforts should prioritize the retention of mature trees, park-like greenery, and structurally complex vegetation within monastery and church grounds. The removal of old trees and simplification of vegetation structure substantially reduce habitat quality for birds.

Several limitations should be acknowledged. First, the use of short-duration point counts and two visits per station may have limited the detectability of less common or cryptic species. Second, our operationalisation of sacred natural sites focused on the presence of monasteries and historical land-use legacy, without explicitly accounting for contemporary socio-cultural practices or management intensity, nor for other ecosystem components beyond birds. Lastly, we did not apply methods accounting for detectability (e.g., N-mixture or distance sampling models), which could provide more robust inference on abundance and distribution. Future studies incorporating such approaches, as well as multi-species and socio-ecological perspectives, would further strengthen inference in these systems.

## 5. Conclusions

Our study shows that the composition of breeding bird assemblages was not significantly influenced by Cistercian legacy. Instead, bird communities were primarily shaped by habitat-related factors. Assemblages varied along a gradient from species-poor farmland to more heterogeneous environments, such as urban areas and grasslands, which offer a greater diversity of microhabitats. These results indicate that habitat complexity and heterogeneity, characteristic of ecologically richer landscapes, are the main drivers of bird assemblage diversity, outweighing monastic legacy.

## Data Availability

The original contributions presented in this study are included in the Supplementary Materials. Further inquiries can be directed to the corresponding author.

## Supplementary Materials

The following supporting information can be downloaded at https://www.mdpi.com/article/10.3390/ani16101534/s1, Table S1: Average habitat composition of different plot types; Table S2: Breeding-bird species counts in Cistercian, Control, and Post-Cistercian plots, with totals and proportional composition, arranged starting from the most abundant species; Table S3: Comparison of breeding bird assemblages between monastery garden/grounds and other station points (abundance and composition), with totals per species; Table S4: Mixed-effects model for Shannon diversity (H’); Table S5: Mixed-effects model for rarefied species richness; Figure S1: Correlation plot of continuous variables.
