# Supplementary material for: Habitat Structure Outweighs Monastic Legacy in Shaping Bird Assemblages
Source: Animals (Basel). 2026 May 17;16(10):1534. doi: 10.3390/ani16101534 (PMC13203236; doi:10.3390/ani16101534)
Supplement: Supplementary file 1 [file animals-16-01534-s001.zip › animals-4269523-supplementary.pdf]

Habitat Structure Outweighs Monastic Legacy in Shaping Bird Assemblages

Supplementary Materials

Table S1. Average habitat composition of different plot types. N - number of stations.

Plot	N	Arable land	Forest	Meadow and grassland	Heterogenous agricultural areas	Urban fabric	Inland wetlands	Inland waters
Cistercian	84	34.4	7.25	22.9	8.28	25.7	0.71	0.724
Control	80	42	5.82	31.7	4.53	15.3	0.65	0.0319
Post-Cistercian	70	48.4	13.1	7.23	4.04	25.5	0	1.77

Table S2. Breeding-bird species counts in Cistercian, Control, and Post-Cistercian plots, with totals and proportional composition, arranged starting from the most abundant species.

n	Species	Cistercian	Control	Post-Cistercian	Total	Composition
1	*Sturnus vulgaris*	287	834	191	1312	17.16%
2	*Alauda arvensis*	134	197	199	530	6.93%
3	*Passer domesticus*	72	150	287	509	6.66%
4	*Columba palumbus*	112	145	154	411	5.38%
5	*Hirundo rustica*	73	129	57	259	3.39%
6	*Emberiza calandra*	69	104	57	230	3.01%
7	*Turdus merula*	75	61	81	217	2.84%
8	*Sylvia atricapilla*	68	65	63	196	2.56%
9	*Streptopelia decaocto*	73	75	47	195	2.55%
10	*Columba livia*	50	47	88	185	2.42%
11	*Grus grus*	37	74	69	180	2.35%
12	*Apus apus*	79	15	76	170	2.22%
13	*Emberiza citrinella*	38	77	34	149	1.95%
14	*Carduelis carduelis*	29	92	17	138	1.81%
15	*Fringilla coelebs*	37	50	38	125	1.64%
16	*Delichon urbicum*	33	53	37	123	1.61%
17	*Cyanistes caeruleus*	18	28	73	119	1.56%

n	Species	Cistercian	Control	Post-Cistercian	Total	Composition
18	*Phylloscopus collybita*	32	27	52	111	1.45%
19	*Phasianus colchicus*	33	30	39	102	1.33%
20	*Parus major*	39	33	28	100	1.31%
21	*Passer montanus*	54	29	15	98	1.28%
22	*Corvus corax*	37	36	24	97	1.27%
23	*Cuculus canorus*	22	38	32	92	1.20%
24	*Luscinia megarhynchos*	22	25	25	72	0.94%
25	*Chloris chloris*	29	32	10	71	0.93%
26	*Curruca communis*	22	33	15	70	0.92%
27	*Pica pica*	21	22	27	70	0.92%
28	*Oriolus oriolus*	20	33	17	70	0.92%
29	*Motacilla flava*	16	24	28	68	0.89%
30	*Turdus philomelos*	23	22	23	68	0.89%
31	*Coloeus monedula*	38	23	4	65	0.85%
32	*Anas platyrhynchos*	24	27	12	63	0.82%
33	*Linaria cannabina*	29	22	10	61	0.80%
34	*Motacilla alba*	19	30	10	59	0.77%
35	*Phoenicurus phoenicurus*	27	8	22	57	0.75%
36	*Phoenicurus ochruros*	23	22	8	53	0.69%
37	*Corvus cornix*	9	20	22	51	0.67%
38	*Phylloscopus trochilus*	6	33	8	47	0.61%
39	*Serinus serinus*	24	9	13	46	0.60%
40	*Curruca curruca*	17	14	12	43	0.56%
41	*Turdus pilaris*	7	13	22	42	0.55%
42	*Upupa epops*	8	26	5	39	0.51%
43	*Anser anser*	17	19	2	38	0.50%
44	*Chroicocephalus ridibundus*	29	2	7	38	0.50%

n	Species	Cistercian	Control	Post-Cistercian	Total	Composition
45	*Troglodytes troglodytes*	9	6	19	34	0.44%
46	*Dendrocopos major*	10	11	9	30	0.39%
47	*Acrocephalus arundinaceus*	7	20	3	30	0.39%
48	*Coccothraustes coccothraustes*	15	12	2	29	0.38%
49	*Lanius collurio*	8	15	5	28	0.37%
50	*Garrulus glandarius*	14	12	2	28	0.37%
51	*Saxicola rubicola*	3	13	10	26	0.34%
52	*Acrocephalus palustris*	10	10	6	26	0.34%
53	*Lullula arborea*	3	20	1	24	0.31%
54	*Larus canus*	0	0	23	23	0.30%
55	*Ciconia ciconia*	10	7	5	22	0.29%
56	*Columba oenas*	1	14	7	22	0.29%
57	*Buteo buteo*	5	11	5	21	0.27%
58	*Galerida cristata*	6	3	10	19	0.25%
59	*Phalacrocorax carbo*	9	8	2	19	0.25%
60	*Falco tinnunculus*	5	10	4	19	0.25%
61	*Vanellus vanellus*	0	15	3	18	0.24%
62	*Certhia brachydactyla*	5	2	10	17	0.22%
63	*Circus aeruginosus*	4	6	6	16	0.21%
64	*Corvus frugilegus*	6	9	1	16	0.21%
65	*Acrocephalus schoenobaenus*	8	5	3	16	0.21%
66	*Ardea cinerea*	7	6	2	15	0.20%
67	*Sitta europaea*	1	4	10	15	0.20%
68	*Emberiza schoeniclus*	5	7	3	15	0.20%
69	*Erithacus rubecula*	4	3	8	15	0.20%
70	*Aegithalos caudatus*	0	10	2	12	0.16%
71	*Regulus ignicapilla*	5	1	6	12	0.16%

n	Species	Cistercian	Control	Post-Cistercian	Total	Composition
72	*Picus viridis*	5	2	4	11	0.14%
73	*Sylvia borin*	2	6	3	11	0.14%
74	*Saxicola rubetra*	0	7	4	11	0.14%
75	*Cygnus olor*	4	5	2	11	0.14%
76	*Oenanthe oenanthe*	2	2	4	8	0.10%
77	*Haliaeetus albicilla*	6	0	2	8	0.10%
78	*Milvus milvus*	2	2	4	8	0.10%
79	*Jynx torquilla*	2	5	1	8	0.10%
80	*Hippolais icterina*	3	3	2	8	0.10%
81	*Locustella luscinioides*	5	1	1	7	0.09%
82	*Poecile montanus*	3	4	0	7	0.09%
83	*Coturnix coturnix*	3	2	2	7	0.09%
84	*Lanius excubitor*	3	2	2	7	0.09%
85	*Acrocephalus scirpaceus*	2	2	3	7	0.09%
86	*Ardea alba*	6	0	0	6	0.08%
87	*Dryocopus martius*	3	3	0	6	0.08%
88	*Locustella fluviatilis*	1	3	2	6	0.08%
89	*Crex crex*	1	4	0	5	0.07%
90	*Anthus pratensis*	2	2	1	5	0.07%
91	*Locustella naevia*	2	1	2	5	0.07%
92	*Botaurus stellaris*	1	0	3	4	0.05%
93	*Lophophanes cristatus*	3	0	1	4	0.05%
94	*Curruca nisoria*	1	3	0	4	0.05%
95	*Mareca strepera*	0	4	0	4	0.05%
96	*Chlidonias niger*	2	2	0	4	0.05%
97	*Periparus ater*	1	1	2	4	0.05%
98	*Fulica atra*	2	1	1	4	0.05%
99	*Larus argentatus* s.l.	0	1	3	4	0.05%

n	Species	Cistercian	Control	Post-Cistercian	Total	Composition
100	*Riparia riparia*	0	3	0	3	0.04%
101	*Anas crecca*	0	3	0	3	0.04%
102	*Perdix perdix*	3	0	0	3	0.04%
103	*Mergus merganser*	3	0	0	3	0.04%
104	*Prunella modularis*	3	0	0	3	0.04%
105	*Dryobates minor*	1	1	0	2	0.03%
106	*Accipiter nisus*	1	0	1	2	0.03%
107	*Gallinago gallinago*	0	2	0	2	0.03%
108	*Larus cachinnans*	0	2	0	2	0.03%
109	*Larus fuscus*	2	0	0	2	0.03%
110	*Muscicapa striata*	1	0	1	2	0.03%
111	*Tachybaptus ruficollis*	1	1	0	2	0.03%
112	*Certhia familiaris*	1	0	1	2	0.03%
113	*Thinornis dubius*	0	2	0	2	0.03%
114	*Luscinia luscinia*	2	0	0	2	0.03%
115	*Circus cyaneus*	0	1	0	1	0.01%
116	*Spinus spinus*	0	1	0	1	0.01%
117	*Picus canus*	1	0	0	1	0.01%
118	*Dendrocoptes medius*	0	0	1	1	0.01%
119	*Astur gentilis*	0	1	0	1	0.01%
120	*Gallinula chloropus*	1	0	0	1	0.01%
121	*Tringa totanus*	0	1	0	1	0.01%
122	*Numenius arquata*	0	1	0	1	0.01%
123	*Regulus regulus*	0	0	1	1	0.01%
124	*Turdus viscivorus*	0	1	0	1	0.01%
125	*Podiceps cristatus*	1	0	0	1	0.01%
126	*Remiz pendulinus*	1	0	0	1	0.01%
127	*Sterna hirundo*	0	1	0	1	0.01%

n	Species	Cistercian	Control	Post-Cistercian	Total	Composition
128	*Pernis apivorus*	1	0	0	1	0.01%
129	*Streptopelia turtur*	0	1	0	1	0.01%
130	*Asio otus*	0	0	1	1	0.01%
131	*Alcedo atthis*	1	0	0	1	0.01%
132	*Anthus trivialis*	0	0	1	1	0.01%
133	*Phylloscopus sibilatrix*	0	1	0	1	0.01%

Table S3. Comparison of breeding bird assemblages between monastery garden/grounds and other station points (abundance and composition), with totals per species.

		Monastery		Other		
	Species	N	Composition	N	Composition	Total
1.	*Columba livia*	30	12.40%	155	2.09%	185
2.	*Columba palumbus*	26	10.74%	385	5.20%	411
3.	*Sturnus vulgaris*	24	9.92%	1288	17.40%	1312
4.	*Streptopelia decaocto*	10	4.13%	185	2.50%	195
5.	*Alauda arvensis*	9	3.72%	521	7.04%	530
6.	*Sylvia atricapilla*	8	3.31%	188	2.54%	196
7.	*Fringilla coelebs*	8	3.31%	117	1.58%	125
8.	*Parus major*	7	2.89%	93	1.26%	100
9.	*Passer domesticus*	6	2.48%	503	6.80%	509
10.	*Apus apus*	6	2.48%	164	2.22%	170
11.	*Carduelis carduelis*	6	2.48%	132	1.78%	138
12.	*Phylloscopus collybita*	6	2.48%	105	1.42%	111
13.	*Chloris chloris*	6	2.48%	65	0.88%	71
14.	*Serinus serinus*	6	2.48%	40	0.54%	46
15.	*Turdus merula*	5	2.07%	212	2.86%	217
16.	*Delichon urbicum*	5	2.07%	118	1.59%	123
17.	*Corvus corax*	5	2.07%	92	1.24%	97

	Species	Monastery N	Monastery Composition	Other N	Other Composition	Total
18.	*Turdus pilaris*	5	2.07%	37	0.50%	42
19.	*Turdus philomelos*	4	1.65%	64	0.86%	68
20.	*Falco tinnunculus*	4	1.65%	15	0.20%	19
21.	*Hirundo rustica*	3	1.24%	256	3.46%	259
22.	*Emberiza calandra*	3	1.24%	227	3.07%	230
23.	*Phoenicurus phoenicurus*	3	1.24%	54	0.73%	57
24.	*Phoenicurus ochruros*	3	1.24%	50	0.68%	53
25.	*Cyanistes caeruleus*	2	0.83%	117	1.58%	119
26.	*Phasianus colchicus*	2	0.83%	100	1.35%	102
27.	*Cuculus canorus*	2	0.83%	90	1.22%	92
28.	*Curruca communis*	2	0.83%	68	0.92%	70
29.	*Pica pica*	2	0.83%	68	0.92%	70
30.	*Motacilla flava*	2	0.83%	66	0.89%	68
31.	*Anas platyrhynchos*	2	0.83%	61	0.82%	63
32.	*Motacilla alba*	2	0.83%	57	0.77%	59
33.	*Ciconia ciconia*	2	0.83%	20	0.27%	22
34.	*Ardea cinerea*	2	0.83%	13	0.18%	15
35.	*Regulus ignicapilla*	2	0.83%	10	0.14%	12
36.	*Poecile montanus*	2	0.83%	5	0.07%	7
37.	*Grus grus*	1	0.41%	179	2.42%	180
38.	*Oriolus oriolus*	1	0.41%	69	0.93%	70
39.	*Coloeus monedula*	1	0.41%	64	0.86%	65
40.	*Linaria cannabina*	1	0.41%	60	0.81%	61
41.	*Curruca curruca*	1	0.41%	42	0.57%	43
42.	*Upupa epops*	1	0.41%	38	0.51%	39
43.	*Chroicocephalus ridibundus*	1	0.41%	37	0.50%	38

		Monastery		Other		
	Species	N	Composition	N	Composition	Total
44.	*Acrocephalus arundinaceus*	1	0.41%	29	0.39%	30
45.	*Coccothraustes coccothraustes*	1	0.41%	28	0.38%	29
46.	*Buteo buteo*	1	0.41%	20	0.27%	21
47.	*Galerida cristata*	1	0.41%	18	0.24%	19
48.	*Certhia brachydactyla*	1	0.41%	16	0.22%	17
49.	*Picus viridis*	1	0.41%	10	0.14%	11
50.	*Hippolais icterina*	1	0.41%	7	0.09%	8
51.	*Coturnix coturnix*	1	0.41%	6	0.08%	7
52.	*Acrocephalus scirpaceus*	1	0.41%	6	0.08%	7
53.	*Dryocopus martius*	1	0.41%	5	0.07%	6
54.	*Prunella modularis*	1	0.41%	2	0.03%	3
55.	*Podiceps cristatus*	1	0.41%	0	0.00%	1
56.	*Pernis apivorus*	1	0.41%	0	0.00%	1
57.	*Emberiza citrinella*	0	0.00%	149	2.01%	149
58.	*Passer montanus*	0	0.00%	98	1.32%	98
59.	*Luscinia megarhynchos*	0	0.00%	72	0.97%	72
60.	*Corvus corone*	0	0.00%	51	0.69%	51
61.	*Phylloscopus trochilus*	0	0.00%	47	0.63%	47
62.	*Anser anser*	0	0.00%	38	0.51%	38
63.	*Troglodytes troglodytes*	0	0.00%	34	0.46%	34
64.	*Dendrocopos major*	0	0.00%	30	0.41%	30
65.	*Lanius collurio*	0	0.00%	28	0.38%	28
66.	*Garrulus glandarius*	0	0.00%	28	0.38%	28
67.	*Saxicola rubicola*	0	0.00%	26	0.35%	26
68.	*Acrocephalus palustris*	0	0.00%	26	0.35%	26
69.	*Lullula arborea*	0	0.00%	24	0.32%	24

		Monastery		Other		
	Species	N	Composition	N	Composition	Total
70.	*Larus canus*	0	0.00%	23	0.31%	23
71.	*Columba oenas*	0	0.00%	22	0.30%	22
72.	*Phalacrocorax carbo*	0	0.00%	19	0.26%	19
73.	*Vanellus vanellus*	0	0.00%	18	0.24%	18
74.	*Circus aeruginosus*	0	0.00%	16	0.22%	16
75.	*Corvus frugilegus*	0	0.00%	16	0.22%	16
76.	*Acrocephalus schoenobaenus*	0	0.00%	16	0.22%	16
77.	*Sitta europaea*	0	0.00%	15	0.20%	15
78.	*Emberiza schoeniclus*	0	0.00%	15	0.20%	15
79.	*Erithacus rubecula*	0	0.00%	15	0.20%	15
80.	*Aegithalos caudatus*	0	0.00%	12	0.16%	12
81.	*Sylvia borin*	0	0.00%	11	0.15%	11
82.	*Saxicola rubetra*	0	0.00%	11	0.15%	11
83.	*Cygnus olor*	0	0.00%	11	0.15%	11
84.	*Oenanthe oenanthe*	0	0.00%	8	0.11%	8
85.	*Haliaeetus albicilla*	0	0.00%	8	0.11%	8
86.	*Milvus milvus*	0	0.00%	8	0.11%	8
87.	*Jynx torquilla*	0	0.00%	8	0.11%	8
88.	*Locustella luscinioides*	0	0.00%	7	0.09%	7
89.	*Lanius excubitor*	0	0.00%	7	0.09%	7
90.	*Ardea alba*	0	0.00%	6	0.08%	6
91.	*Locustella fluviatilis*	0	0.00%	6	0.08%	6
92.	*Crex crex*	0	0.00%	5	0.07%	5
93.	*Anthus pratensis*	0	0.00%	5	0.07%	5
94.	*Locustella naevia*	0	0.00%	5	0.07%	5
95.	*Botaurus stellaris*	0	0.00%	4	0.05%	4
96.	*Lophophanes cristatus*	0	0.00%	4	0.05%	4

	Species	Monastery N	Monastery Composition	Other N	Other Composition	Total
97.	*Curruca nisoria*	0	0.00%	4	0.05%	4
98.	*Mareca strepera*	0	0.00%	4	0.05%	4
99.	*Chlidonias niger*	0	0.00%	4	0.05%	4
100.	*Periparus ater*	0	0.00%	4	0.05%	4
101.	*Fulica atra*	0	0.00%	4	0.05%	4
102.	*Larus argentatus* s.l.	0	0.00%	4	0.05%	4
103.	*Riparia riparia*	0	0.00%	3	0.04%	3
104.	*Anas crecca*	0	0.00%	3	0.04%	3
105.	*Perdix perdix*	0	0.00%	3	0.04%	3
106.	*Mergus merganser*	0	0.00%	3	0.04%	3
107.	*Dryobates minor*	0	0.00%	2	0.03%	2
108.	*Accipiter nisus*	0	0.00%	2	0.03%	2
109.	*Gallinago gallinago*	0	0.00%	2	0.03%	2
110.	*Larus cachinnans*	0	0.00%	2	0.03%	2
111.	*Larus fuscus*	0	0.00%	2	0.03%	2
112.	*Muscicapa striata*	0	0.00%	2	0.03%	2
113.	*Tachybaptus ruficollis*	0	0.00%	2	0.03%	2
114.	*Certhia familiaris*	0	0.00%	2	0.03%	2
115.	*Thinornis dubius*	0	0.00%	2	0.03%	2
116.	*Luscinia luscinia*	0	0.00%	2	0.03%	2
117.	*Circus cyaneus*	0	0.00%	1	0.01%	1
118.	*Spinus spinus*	0	0.00%	1	0.01%	1
119.	*Picus canus*	0	0.00%	1	0.01%	1
120.	*Dendrocoptes medius*	0	0.00%	1	0.01%	1
121.	*Astur gentilis*	0	0.00%	1	0.01%	1
122.	*Gallinula chloropus*	0	0.00%	1	0.01%	1
123.	*Tringa totanus*	0	0.00%	1	0.01%	1

	Species	Monastery N	Monastery Composition	Other N	Other Composition	Total
124.	*Numenius arquata*	0	0.00%	1	0.01%	1
125.	*Regulus regulus*	0	0.00%	1	0.01%	1
126.	*Turdus viscivorus*	0	0.00%	1	0.01%	1
127.	*Remiz pendulinus*	0	0.00%	1	0.01%	1
128.	*Sterna hirundo*	0	0.00%	1	0.01%	1
129.	*Streptopelia turtur*	0	0.00%	1	0.01%	1
130.	*Asio otus*	0	0.00%	1	0.01%	1
131.	*Alcedo atthis*	0	0.00%	1	0.01%	1
132.	*Anthus trivialis*	0	0.00%	1	0.01%	1
133.	*Phylloscopus sibilatrix*	0	0.00%	1	0.01%	1

Table S4. Mixed-effects model for Shannon diversity (H').

Intercept	2.231	0.088	3.4	25.38	<0.001
Plot (Control)	0.096	0.124	3.4	0.77	0.491
Plot (Post-Cistercian)	-0.012	0.074	15.2	-0.17	0.869
Monastery-direct (Y)	-0.046	0.124	223.5	-0.37	0.710
Arable → Urban (PC1)	0.045	0.020	150.1	2.28	0.024
Arable → Grassland (PC2)	0.084	0.022	210.9	3.85	<0.001
Urban → Grassland Forest (PC3)	0.027	0.023	215.1	1.22	0.225
Heterogeneous agriculture (PC4)	0.117	0.023	223.1	5.04	<0.001

Table S5. Mixed-effects model for rarefied species richness.

Intercept	5.402	0.119	5.1	45.30	<0.001
Plot (Control)	-0.038	0.217	3.4	-0.18	0.870
Plot (Post-Cistercian)	-0.068	0.190	5.5	-0.36	0.735
Monastery-direct (Y)	-0.014	0.277	221.2	-0.05	0.959
Arable → Urban (PC1)	0.047	0.049	83.4	0.96	0.338
Arable → Grassland (PC2)	0.179	0.052	125.7	3.47	<0.001
Urban → Grassland Forest (PC3)	0.105	0.051	174.7	2.05	0.042
Heterogeneous agriculture (PC4)	0.280	0.053	223.5	5.25	<0.001

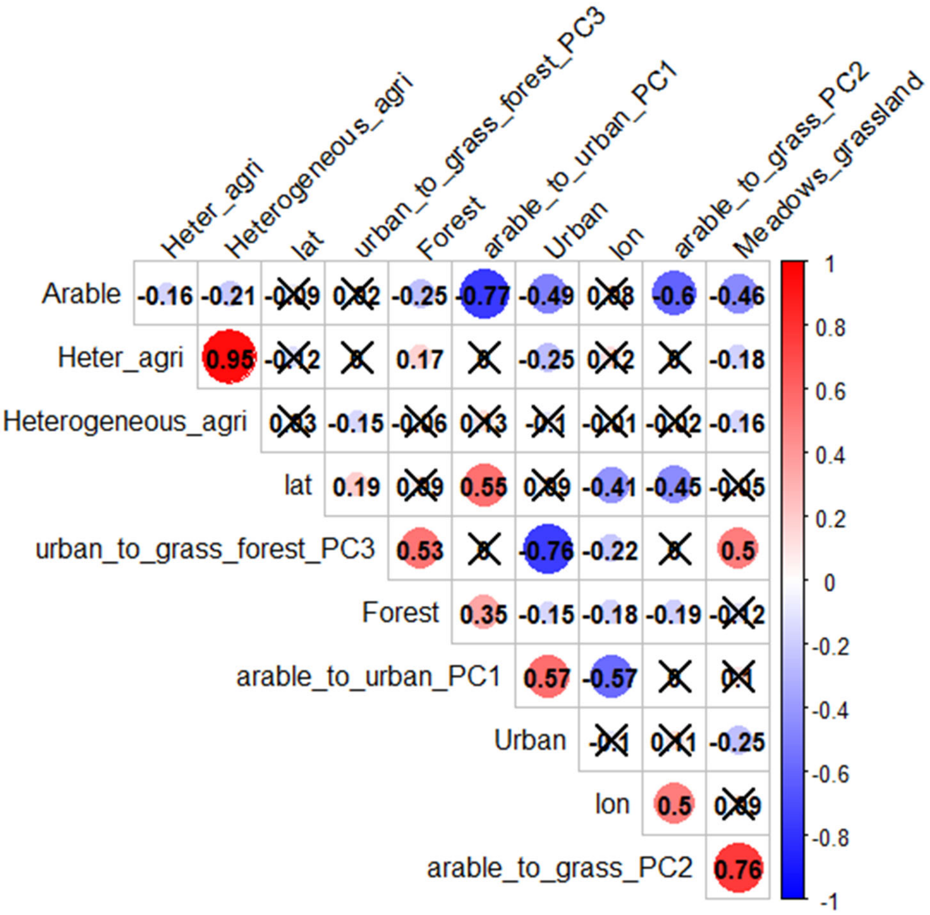

Figure. S1. Correlation plot of continuous variables.
